# A prospective study to compare the safety and efficacy of toric intra-ocular lens vs. opposite clear corneal incision in patients undergoing phacoemulsification for age-related cataract with pre-existing corneal astigmatism

**DOI:** 10.22336/rjo.2025.13

**Published:** 2025

**Authors:** Sandeep Gupta, Nidhi Kalra, Ankita Singh, Avinash Mishra, Jaya Kaushik

**Affiliations:** 1 Department of Ophthalmology, Military Hospital Suratgarh, Rajasthan, India; 2 Department of Ophthalmology, Base Hospital Guwahati, Assam, India; 3 Department of Ophthalmology, Military Hospital, Bathinda, Punjab, India; 4 Department of Ophthalmology, Military Hospital Jalandhar, Punjab, India; 5 Department of Ophthalmology, Command Hospital (Lucknow), India

**Keywords:** pre-existing astigmatism, Toric Intra Ocular Lens, Opposite Clear Corneal Incision, phacoemulsification, PEA = Preexisting astigmatism, SIA = surgically induced astigmatism, OCCI = opposite clear corneal incision, CCI = clear corneal incision, LASIK = laser in situ keratomileusis, IOLs = intraocular lenses, BCVA = best-corrected visual acuity, UCVA = uncorrected visual acuity

## Abstract

**Purpose:**

This study aimed to compare the safety and efficacy of toric intra ocular lens (IOL) implantation vs. opposite clear corneal incision (OCCI) during cataract surgery to correct preexisting corneal astigmatism in patients with age related cataract by comparing the postoperative residual astigmatism, the uncorrected distance visual acuity and the adverse effects following both the procedures.

**Methods:**

A pilot prospective study was conducted at a tertiary hospital among patients undergoing cataract surgery over two years. A total of 150 eyes with pre-existing corneal astigmatism between 1 to 2.5 D were divided into two groups of 75 each by permuted block randomization. Group A underwent implantation of *AcrySof Toric IOLSN6AT4* (Alcon Labs, Fort Worth, Tx), and Group B underwent implantation of normal non-toric aspheric *AcrySof IOL SN60WF with* OCCI on steep axis. Both groups were followed up for 24 weeks.

**Results:**

In the OCCI Group, the difference between mean K1-K2 preoperative and 12 weeks postoperative (mean ± SD) of patients was 1.55 ± 0.17D. In the TORIC IOL Group, the difference between the mean K1-K2 preoperative and 12-week postoperative values (mean ± SD) for patients was 0.53 ± 0.11. The difference in mean K1-K2 pre-operatively and at 12 weeks was statistically significant for both groups (p < 0.0001). There was a significant reduction in corneal astigmatism in the OCCI group at 01 week.

**Discussion:**

In our study, the difference in mean K1-K2 at 1 week between the two Groups was statistically significant (p < 0.0001), as OCCI acts directly on the corneal plane, whereas Toric IOLs reduce astigmatism at the lens plane without affecting corneal curvature. There was a significant reduction in corneal astigmatism in the OCCI group at 1 week. This reduction continued to stabilize at 12 weeks. The corneal astigmatism remained stable in the post-operative period in the Toric IOL group. The decrease in astigmatism was more significant in the toric IOL group than in the OCCI group.

**Conclusions:**

These results demonstrate a similar efficacy of OCCI in reducing corneal astigmatism of up to 1.5 D compared to Toric IOLs. The uncorrected visual acuity was superior in the Toric IOL group. However, this difference was reduced significantly by 12 weeks.

## Introduction

Preexisting astigmatism (PEA) is one of the most common causes of low vision following cataract surgery, with 70% of the general population having astigmatism of more than one diopter (D), and 30% of patients selected for cataract surgery requiring correction of astigmatism to achieve a spectacle-free life [1]. The visual outcome after cataract surgery is significantly affected by both pre-existing astigmatism (PEA) and surgically induced astigmatism (SIA) [2].

Several methods have been employed for this purpose, including changing the size and site of the incision, using corneal or limbal relaxing incisions, applying opposite clear corneal incision (OCCI) on the steep axis, implantation of toric IOLs, and laser in situ keratomileusis (LASIK) [3-8] - all the above-mentioned measures aimed to achieve acceptable uncorrected visual acuity (UCVA) and improve patient satisfaction.

Making a small, clear corneal incision (CCI) on the steep axis can reduce PEA ranging from 0.25-0.75D [9]. Taking a clue from on-axis incision, it is reasoned that making an additional identical incision of 180° opposite to the on-axis incision, which is also called paired opposite clear corneal incision (OCCI), would enhance the flattening effect on the cornea and hence would be further helpful in reducing PEA [10]. This is a straightforward procedure that does not require any additional skills or instruments. Lever and Dahan first applied this technique, reporting a mean astigmatism correction of 2.06 D [5]. The current gold standard for correcting PEA is the use of toric intraocular lenses (IOLs) [1,1-1,3]. They are considered for patients with more than 1.0 D of regular corneal astigmatism who desire spectacle-free distance vision after cataract surgery. Unlike OCCI, which functions by correcting preexisting corneal astigmatism at the corneal surface, toric IOLs compensate for corneal astigmatism in the IOL plane [14-16].

Toric IOLs are expensive and may be inappropriate in resource-constrained settings and developing countries, potentially paving the way for relatively inexpensive procedures to correct PEA. Since there is limited data and comparison from structured studies between commonly performed Toric IOLs and the potentially more straightforward, repeatable, and cost-effective option of OCCI, this study was designed to scientifically analyze the two techniques, thereby filling the existing knowledge gap.

## Materials and methods

After obtaining clearance from the institute’s ethics committee, by the ethical principles outlined in the Declaration of Helsinki (IEC No. 15965, AFMC, Pune), the study was conducted at a tertiary hospital. The subjects enrolled were sourced from those attending the Ophthalmology OPD and were planned to undergo cataract surgery. It was a pilot prospective randomized clinical observational study.

### Inclusion criteria

All consecutive eyes with age-related cataract and pre-existing corneal astigmatism between 1 to 2.5 D who were scheduled for cataract surgery during the study period were included in this study.

### Exclusion criteria

The following patients were excluded from the study: all cases with preexisting corneal astigmatism <1 D or > 2.5 D, patients having anterior segment disorders affecting corneal curvature e.g. corneal infiltration, scarring, ectasia, pterygium and high irregular astigmatism, any intra operative complication affecting corneal astigmatism or visual prognosis and patients having any posterior segment disorders affecting visual prognosis.

### Methodology and sample

A total of 150 eyes were recruited for evaluation and were divided into two groups of 75 each by permuted block randomization. Group A underwent cataract surgery with implantation of *AcrySof Toric IOLSN6AT4* (Alcon Labs, Fort Worth, Tx). Group B underwent cataract surgery with implantation of normal non toric aspheric *AcrySof IOL SN60WF* (Alcon Labs, Fort Worth, Tx) and OCCI on steep axis. Both groups were followed up for 24 weeks.

Complete ophthalmological assessment was done before surgery and the following parameters were noted: Uncorrected visual acuity (UCVA) by LogMAR charts, refraction using streak retinoscope without cycloplegia, best corrected visual acuity (BCVA) by LogMAR charts, Keratometric K1 (dioptric power of flatter corneal meridian) and K2 (dioptric power of steeper corneal meridian) readings using optical biometer and intraocular pressure measurement by non-contact tonometry. Slit lamp examination of anterior segment, grading of nuclear sclerosis according to Lens Opacities Classification System III and fundus examination by indirect ophthalmoscopy was done. IOL power was calculated by optical biometry using IOL master (Carl Zeiss 500, USA) using Barrett’s formula. Written informed consent was obtained from each patient. One day before surgery, all patients were started on broad-spectrum antibiotic eye drops (Moxifloxacin), one drop four times a day in both eyes.

### Outcome measures

Primary Outcome Measures: postoperative residual astigmatism at 3 months.

Secondary Outcome Measures: uncorrected distance visual acuity (UDVA) at 3 months post-operatively, adverse effects, if any, and postoperative IOL rotation, if any.

### Surgical technique

**Fig. 1:**
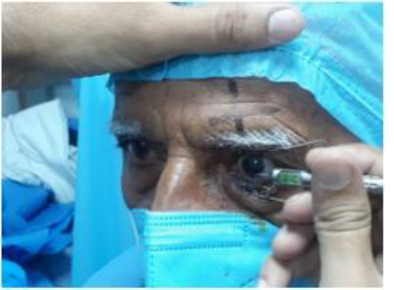
Standard Axis marking

The surgeries were performed under topical anaesthesia (0.5% proparacaine) by a single surgeon. On the day of surgery, the pupil of the patients was dilated by the instillation of Tropicamide 1% with phenylephrine 2.5% every 10 minutes for 30 minutes. Standard axis marking was performed before surgery in all cases using a bubble marker in the sitting position (**Fig. 1**).

**Fig. 2:**
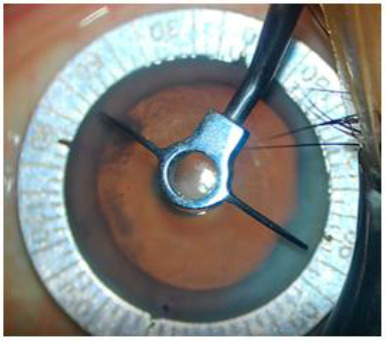
Steep/Toric Axis marking on Table

Under strict aseptic precautions, after dressing and draping, a solid blade-guarded speculum was applied. Povidone iodine 5% solution was instilled and thoroughly washed after being retained for 3 minutes. The Steep/Toric IOL axis was marked using a proprietary Toric axis marker by Alcon Labs, Fort Worth, Tx. First, two side ports, 0.9 mm in size, were created with an MVR blade. Hydroxyl propyl methyl cellulose 2% was introduced into the anterior chamber. Continuous curvilinear capsulorhexis was performed by a bent tipped 26G cystotome. The main incision was a 2.8 mm biplanar, clear corneal incision on the steep axis in all selected cases in the OCCI group and at the standard location as per the Toric IOL calculation in the Toric IOL group. Hydro dissection and hydro delineation were performed to achieve free rotation of the nucleus (**Fig. 2**).

**Fig. 3:**
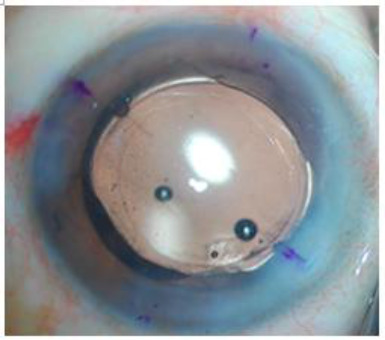
Toric IOL placed at pre-determined Axis

Phacoemulsification was performed using the Stellaris phaco system with CSS software (Bausch & Lomb, NY, USA). After irrigation and aspiration of cortical matter by co-axial technique, the capsular bag was filled with viscoelastic and in Group A, *AcrySof Toric IOLSN6AT4* (Alcon Labs, Fort Worth, Tx) and in Group B, normal non toric aspheric *AcrySof IOL SN60WF* (Alcon Labs, Fort Worth, Tx) was implanted in all patients. After proper lens placement, an additional 2.8 mm self-sealing clear corneal incision was made in the 2nd group (Paired OCCI) 1800 opposite to the main incision. In the Toric IOL group, the axis of the IOL was matched with a pre-determined axis of implantation before wound closure (**Fig. 3**).

Finally, the viscoelastic substance was removed, and the corneal wound was closed by stromal hydration with a balanced salt solution. The eye patch was removed after 3 hours on the same day of surgery, and patients were prescribed broad-spectrum antibiotic and steroid eye drops (Moxifloxacin + Dexamethasone) 10 times a day, tapered over the next four weeks. On the first postoperative day, patients were examined to rule out any surgical complications. Postoperative follow up was done for 12 weeks, which consisted of four visits: on 1^st^ day, 1^st^ week, 4^th^ week and 12^th^ week after surgery: K1(dioptric power of flatter corneal meridian), K2 (dioptric power of steeper corneal meridian) and UCVA (LogMAR) readings were noted on the 2^nd^, 3^rd^ and 4^th^ visits. Any complications and significant rotation of the Toric IOL were also pointed out at all visits.

### Statistical analysis

The statistical software SPSS version 20 was used for the analysis. An alpha level of 5% was used, i.e., if any p-value was less than 0.05, it was considered statistically significant.

Data were recorded on a predesigned pro forma document and managed in an Excel spreadsheet.

Continuous variables were expressed as Mean ± Standard Deviation and compared across groups using unpaired t test/One Way ANOVA for data following normal distribution and Mann-Whitney U test/Kruskal Wallis Test for data not following normal distribution. Comparison over time was performed using a paired t-test for data that followed a normal distribution and a Wilcoxon signed-rank test for data that did not follow a normal distribution.

## Results

In our study, 75 patients (50.0%) were assigned to the OCCI group, and 75 patients (50.0%) were assigned to the TORIC IOL group.

### Age and sex distribution

In the OCCI Group, the mean Age (mean ± SD) of patients was 62.53 ± 6.66 years. In the TORIC IOL Group, the mean Age (mean ± SD) of patients was 64.05 ± 7.49 years. In the OCCI Group, 3 (4.0%) patients were 40-50 years old, 25 (33.3%) patients were 51-60 years old, 39 (52.0%) patients were 61-70 years old, and 8 (10.7%) patients were 71-80 years old. In TORIC IOL Group, 4 (5.3%) patients were 40-50 years old, 14 (18.7%) patients were 51-60 years old, 47 (62.7%) patients were 61-70 years old, 8 (10.7%) patients were 71-80 years old and 2 (2.7%) patients were >81 years old.

In the OCCI Group, 29 (38.7%) patients were female, and 46 (61.3%) patients were male. In the TORIC IOL Group, 23 (30.7%) patients were female, and 52 (69.3%) patients were male. The association between sex and group was not statistically significant (p = 0.3032).

*Preoperative Vision and Keratometry*

In the OCCI Group, the mean UCVA (LogMAR) preoperatively was 0.78 ± 0.29. In the TORIC IOL Group, the mean UCVA (LogMAR) preoperatively (mean ± SD) was 0.84 ± 0.23. The difference in mean UCVA (LogMAR) between the preoperative groups was not statistically significant (p = 0.2180). In the OCCI Group, the mean K1-K2 difference preoperatively (mean ± SD) was 1.94 ± 0.12 D. In the TORIC IOL Group, the mean K1-K2 difference preoperatively (mean ± SD.) was 1.98 ± 0.08 D. The difference of mean K1-K2 preoperatively with both groups was not statistically significant (p = 0.0614) (**Table 1**).

**Table 1:** Keratometry and UCVA data for both groups preoperatively and postoperatively

Group	Mean UCVA (LogMAR) pre-op (p 0.2180)	Mean K1-K2 pre-op in Diopter (D) p 0.0614	Mean K1-K2 at 1-week post-op in Diopter (D) p 0.0001	Mean UCVA (LogMA) 1-week post-op p 0.0421	MeanK1-K2 at 4-week post-op in Diopter (D) p 0.0001	Mean UCVA (LogMAR) at 4 weeks p 0.0001	Mean K1-K2 at 12 weeks post-op in Diopter (D) p 0.0001	Mean UCVA (Log MAR) at 12 weeks post-op p 0.0014
OCCI	0.78 + 0.29	1.94 + 0.12	1.27 + 0.52	0.10 + 0.11	0.41 + 0.13	0.09 + 0.11	0.39 + 0.12	0.04 + 0.10
TORIC IOL	0.84 + 0.23	1.98 + 0.08	1.78 + 0.13	0.0744 + 0.09	1.61 + 0.14	0.01 + 0.05	1.44 + 0.13	0.00 + 0.03

### Postoperative Vision

In the OCCI Group, the mean UCVA (LogMAR) 1 week postoperatively was 0.10 ± 0.11. In the TORIC IOL Group, the mean UCVA (LogMAR) 1 week postoperatively (mean ± SD) was 0.07 ± 0.09. The difference in mean UCVA (LogMAR) at 1 week between the two groups was statistically significant (p = 0.0421). Patients with Toric intraocular lenses (IOLs) had better uncorrected visual acuity compared to those with OCCI at 1 week.

In the OCCI Group, the mean UCVA (LogMAR) at 4 weeks was 0.09 ± 0.11. In the TORIC IOL Group, the mean UCVA (LogMAR) at 4 weeks (mean ± SD) was 0.01 ± 0.05. The difference in mean UCVA (LogMAR) at 4 weeks between the two groups was statistically significant (p < 0.0001). The uncorrected visual superiority of Toric IOLs over OCCI was maintained at 4 weeks. In the OCCI Group, the mean UCVA (Log MAR) at 12 weeks postoperatively was 0.04 ± 0.10. In the TORIC IOL Group, the mean UCVA (Log MAR) at 12 weeks postoperatively (mean ± SD) was 0.00 ± 0.03. The difference in mean UCVA (Log MAR) at 12 weeks between the two groups was statistically significant (p = 0.0014). This also indicates the stabilization of astigmatism resulting from corneal wound healing in both groups.

In the OCCI Group, 36 (48.0%) patients had a UCVA (LogMAR) of 0 at 1 week, 29 (38.7%) patients had a UCVA (LogMAR) of 0.18 at 1 week, and 10 (13.3%) patients had a UCVA (LogMAR) of 0.3 at 1 week. In the TORIC IOL Group, 46 (61.3%) patients had a UCVA (LogMAR) of 0 at 1 week, 26 (34.7%) patients had a UCVA (LogMAR) of 0.18 at 1 week, and 3 (4.0%) patients had a UCVA (LogMAR) of 0.3 at 1 week. The association of UCVA (Log MAR) at 1 week versus the group was not statistically significant (p = 0.0761).

In the OCCI Group, 59 (78.7%) patients had UCVA (LogMAR) 0 at 12 weeks, 12 (16.0%) patients had UCVA (LogMAR) 0.18 at 12 weeks, 2 (2.7%) patients had UCVA (LogMAR) 0.3 at 12 weeks and 2 (2.7%) patients had UCVA (LogMAR) 0.48 at 12 weeks. In the TORIC IOL Group, 72 (96.0%) patients had UCVA (LogMAR) 0 at 12 weeks, and 3 (4.0%) patients had UCVA (LogMAR) 0.3 at 12 weeks. The association between LogMAR at 12 weeks and the group was statistically significant (p = 0.0135).

### Postoperative Keratometry

In the OCCI Group, the mean K1-K2 at 1 week postoperatively (mean ± SD) was 1.27 ± 0.52 D. In the TORIC IOL Group, the mean K1-K2 at 1 week postoperatively (mean ± SD) was 1.78 ± 0.13D. The difference in mean K1-K21 at 1 week between the two groups was statistically significant (p < 0.0001). This was expected, as the OCCIs work at the level of the cornea, reducing corneal astigmatism. In contrast, Toric IOLs work at the lens plane, neutralizing corneal astigmatism without directly decreasing it. In the OCCI Group, the mean K1-K2 preoperative to 1-week postoperative difference (mean ± SD) was 0.67 ± 0.54 D. In the TORIC IOL Group, the mean K1-K2 preop to 1 week postoperative (mean ± SD) difference was 0.19 ± 0.09 D. The difference of mean K1-K2 preoperatively to 1 week postoperatively with both Groups was statistically significant (p < 0.0001). This suggests that OCCI can reduce corneal astigmatism by approximately 0.67 D at approximately one week postoperatively.

In the OCCI Group, the mean K1-K2 difference at 4 weeks postoperatively (mean ± SD) for patients was 0.41 ± 0.13 D. In the TORIC IOL Group, the mean K1-K2 difference at 4 weeks postoperatively (mean ± SD) of patients was 1.61 ± 0.14D. The difference in mean K1-K2 at 4 weeks postoperatively between the two groups was statistically significant (p < 0.0001).

In the OCCI Group, the mean K1-K2 at 12 weeks postoperatively (mean ± SD) was 0.39 ± 0.12 D. In the TORIC IOL Group, the mean K1-K2 at 12 weeks postoperatively (mean ± SD) was 1.44 ± 0.13 D. The difference of mean K1-K2 at 12 weeks within both Groups was statistically significant (p < 0.0001). This change probably represents the ongoing wound modulation and stabilization of corneal astigmatism.

Overall, in the OCCI Group, the mean K1-K2 difference from preoperatively to 12 weeks postoperatively (mean ± SD) was 1.55 ± 0.17 D, signifying a substantial reduction in corneal astigmatism. In the TORIC IOL Group, the mean K1-K2 difference from preoperative to 12 weeks postoperatively (mean ± SD) was 0.53 ± 0.11D. The difference in mean K1-K2 between the preoperative and 12-week postoperative periods for both Groups was statistically significant (p < 0.0001).

### Complications

No complications related to wound construction were noted in either group. There was no case of shallow anterior chamber, hypotony, excessive postoperative reaction, iris prolapse, or endophthalmitis in either group. In the Toric IOL group, rotation of 5° degrees or less from its intended axis was observed in 92% of cases, and rotation of 100 degrees or less in 100% of cases at 3 months postoperatively. IOL repositioning was not required in any of the cases. The most considerable rotation was 9 degrees and was observed in only two eyes. Most IOL rotations occurred in the early postoperative period. Once the anterior and posterior capsules fused, IOL rotation was less frequent at long-term follow-up.

## Discussion

Several techniques have been developed to manage preexisting astigmatism during cataract surgery , aiming to achieve emmetropia and eliminate spectacle dependence. Excimer laser and even femtosecond laser-assisted astigmatic keratotomy can be used postoperatively to correct pre-existing astigmatism in cataract patients; however, this approach is expensive and requires a refractive surgeon [17,18]. Additionally, complications such as loss of best-corrected visual acuity (BCVA), a decentered zone, flap complications, and challenging night vision must be considered [19] . Although astigmatism can be surgically corrected even after cataract surgery, it is more appropriate to combine the two procedures.

Preexisting corneal astigmatism at the time of cataract surgery can be treated by manipulation of cataract incision, limbal relaxing incision, astigmatic keratotomy, or implantation of toric intraocular lenses. The astigmatic modifying effect of the cataract incision site, size, and shape is well documented both in phacoemulsification as well as in manual small incision cataract surgery[20-25].

Lever and Dahan were the first ophthalmologists to introduce the OCCI surgical technique in 2000. They found that a CCI has a small flattening effect on corneal curvature due to the formation of scar tissue where tissue has been separated by incision, which can be used to reduce pre-existing astigmatism[5]. Paired OCCI’s 180 degrees apart on the steepest meridian can enhance the flattening effect. The amount of correction varies but is usually reported to be less than 1.2 D with a single 3.2 mm incision. So, wider incisions or an additional incision have been advocated in cases with higher astigmatism [26,27].

Flattening induced by 3.2 mm is more compared to smaller incisions. Therefore, most studies have used a 3.2 mm incision in OCCI. The mean surgically induced astigmatism in patients with smaller incisions was significantly less than in patients with 3.0 mm incisions [28-31]. As previous studies have reported, OCCIs are self-sealing, pose no added risk, require no additional surgical equipment, and are effective in treating pre-existing astigmatism. However, a second substantial penetrating incision is present, possibly increasing the risk for wound leakage or even infection [32].

**Fig. 4:**
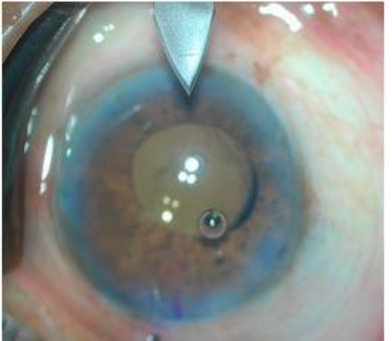
OCCI created after IOL implantation on Table

Reports in the literature indicate a wide variability in age groups among patients undergoing phacoemulsification and toric IOL implantation. Visser et al. analyzed 40 eyes that underwent toric IOL implantation with a mean age of 52.3 + 19.1 years [33]. In a similar study conducted by Venkataraman et al. in South India, the average age of 77 patients undergoing toric IOL implantation was 56 ± 13.88 years [34]. The decrease in the mean age of the patients undergoing phacoemulsification in recent studies can be attributed to the increased safety and reliability of modern-day phacoemulsification techniques (**Fig. 4**).

In earlier studies of OCCIs, the mean reduction in corneal astigmatism ranged from 0.50 to 2.06 D [26-31]. A mean symmetrically induced astigmatism of 2.25 D was reported by Lever and Dahan [5], 2.10 D by Qammar et al. [29], 1.66 D by Ben Simon et al. [35], 1.75 D by Tadros et al. [10], and 1.60 D by Khokhar et al. [31]. The differences between studies are the result of the variety of incision lengths and the amount and type of preexisting astigmatism.

Recently, Binayi et al. evaluated the effect of 4 mm paired incisions on the steep meridian, temporal with the rule (WTR) astigmatism, and vertical against the rule (ATR) along with paired incisions at 180° [26]. The mean surgically induced astigmatism was found to be 1.99 ± 0.9 D. Chiam et al. reported that paired OCCI has a significantly more significant refractive effect on WTR than ATR astigmatism correction. The SIA for ATR astigmatism reduces considerably more than for WTR astigmatism correction at 6 months [27].

In our study, the difference in mean K1-K2 at 1 week between the two Groups was statistically significant (p < 0.0001). This is understandable as OCCI is directly acting on the corneal plane, whereas Toric IOLs reduce astigmatism at the lens plane without affecting corneal curvature. There was a significant reduction in corneal astigmatism in the OCCI group at 1 week. This reduction continued to stabilize at 12 weeks. The corneal astigmatism remained stable in the post-operative period in the Toric IOL group.

Our results are best compared to those reported by Mendicute et al. [14], where 40 eyes of 40 patients were evaluated and the mean refractive cylinder decreased significantly preoperatively to postoperatively 1.75 ± 0.71 D to 0.62 ± 0.46 D in the toric group; 1.61 ± 0.67 D to 0.97 ± 0.51 D in the OCCI group and the mean reduction was 1.13 D in the toric group and 0.64 D in OCCI group.

The mean UCVA was better in the Toric IOL Group at all points. However, the difference decreased over time and became negligible by 12 weeks. The above results were comparable to those of Hoffmann et al., who reported an improvement in mean UCVA from 0.93 to 0.20 in the OCCI group and from 0.41 to 0.09 in the Toric IOL group [36,37].

In the Toric IOL group, rotation of 5^0^ degrees or less from its intended axis was observed in 92%, and of 100 degrees or less in 100% at 3 months postoperatively. The most considerable rotation was 9 degrees and was observed in only two eyes. A one-degree off-axis rotation results in a loss of up to 3.3% of the lens cylinder power [38,39]. Approximately 0.25 D was lost from the axis rotation. Despite this, the reduction in astigmatism was more significant in the toric IOL group than in the OCCI group. The results of the present study are similar to those of Mendicute et al. in terms of astigmatism reduction, visual acuity outcomes, and rotational stability of the toric IOL [36].

## Conclusion

In conclusion, the results of our study demonstrate that both OCCIs and toric IOL implantation are viable surgical options for correcting preexisting corneal astigmatism during cataract surgery. However, toric IOL implantation achieved an enhanced effect over OCCIs in treating preexisting astigmatism. Since there is limited data and comparison from structured studies between commonly performed Toric IOLs and the potentially more straightforward, repeatable, and cost-effective option of OCCI, this study was designed to analyze the two techniques to fill the knowledge gap scientifically.

Future studies with larger samples and longer follow-ups are necessary to evaluate these two approaches for treating astigmatism during cataract surgery. Patients undergoing cataract surgery with preexisting astigmatism of up to 0.75-1.0 diopters (D) can be managed with an on-axis incision only. Patients undergoing cataract surgery with pre-existing astigmatism of less than 1.5 D can be managed equally well with both OCCI and Toric IOLs, depending on the availability of expertise and resources. Beyond 1.5 D pre-existing astigmatism, Toric IOLs are superior and are recommended. However, in a non-affording patient and low-resource setting, OCCIs offer nearly the same benefit as Toric IOLs, especially in astigmatism of up to 1.5 D.
